# Targeting the tumor microenvironment by liposomal Epacadostat in combination with liposomal gp100 vaccine

**DOI:** 10.1038/s41598-023-31007-x

**Published:** 2023-04-10

**Authors:** Sahar Tahaghoghi-Hajghorbani, Mona Yazdani, Amin Reza Nikpoor, Mahdi Hatamipour, Abolghasem Ajami, Mahmoud Reza Jaafari, Ali Badiee, Alireza Rafiei

**Affiliations:** 1 Department of Immunology, School of Medicine, Mazandaran University of Medical Sciences, Sari, Iran; 2 Nanotechnology Research Center, Pharmaceutical Technology Institute, Mashhad University of Medical Sciences, Mashhad, Iran; 3 Department of Immunology, Faculty of Medicine, Hormozgan University of Medical Sciences, Bandar Abbas, Iran; 4 Biotechnology Research Center, Pharmaceutical Technology Institute, Mashhad University of Medical Sciences, Mashhad, Iran; 5 Department of Pharmaceutical Nanotechnology, School of Pharmacy, Mashhad University of Medical Sciences, Mashhad, Iran; 6 Department of Immunology, Molecular and Cell Biology Research Center, School of Medicine, Mazandaran University of Medical Sciences, Sari, Iran

**Keywords:** Cancer immunotherapy, Tumour immunology, Drug discovery, Vaccines

## Abstract

Indoleamine-2,3-dioxygenase (IDO1) pathway has vital role in cancer immune escape and its upregulation leads to immunosuppressive environment which is associated with poor prognosis and progression in various cancers like melanoma. Previously, we showed the antitumoral efficacy of nanoliposomal form of Epacadostat (Lip-EPA), as an IDO1 inhibitor. Herein, we used Lip-EPA as a combination approach with liposomal gp100 (Lip-gp100) anti-cancer vaccine in melanoma model. Here, we showed that B16F10 tumor express IDO1 so using Lip-EPA will enhance the efficacy of vaccine therapy. The biodistribution of ICG-labelled liposomal form of EPA showed the remarkable accumulation of drug at tumor site. In an in vivo study, Lip-EPA enhanced the antitumor efficacy of Lip-gp100 in which the IDO mRNA expression was decreased (~ fourfold) in tumor samples. Also, we identified a significant increase in the number of infiltrated T lymphocytes (p < 0.0001) with enhanced in interferon gamma (IFN-γ) production (p < 0.0001). Additionally, Lip-EPA + Lip-gp100 significantly modulated intratumoral regulatory T cells which altogether resulted in the highest delay in tumor growth (TGD = 56.54%) and increased life span (ILS > 47.36%) in treated mice. Our study demonstrated that novel combination of Lip-EPA and Lip-gp100 was effective treatment with capability of being used in further clinical studies.

## Introduction

Melanoma is the malignant tumor of melanocytes caused by the accumulation of genetic mutations which lead to improper regulation of cellular pathways^1^. Depending on the characteristics of the melanoma (location, stage, and genetic characteristics), treatment options may include surgery, chemotherapy, radiotherapy, photodynamic therapy (PDT), and immunotherapy^2^. While early diagnosis provides the patients with better survival rates, some cases of melanoma remain undiagnosed until late stages. Therefore, the development of new treatments for melanoma is still a priority. The presence of immunosuppressive cells and inhibitory mediators such as the indoleamine deoxygenase (IDO) results in the immunosuppressive nature of the tumor microenvironment in patients with high stages^3–6^.

IDO is a single-chain oxidoreductase which its increased expression has been associated with decreased immune cell infiltration and increased Treg cell infiltration in tumors^7–9^. IDO expression has been reported to increase with the progression of melanoma and is considered as an independent prognostic marker for survival in several cancers^10^. It has been observed that due to IDO inhibition, apoptosis occurs in Treg cells and not in CD8 ^+^ T cells, which play a key role in tumor defense^11^.To date, several IDO inhibitors including 1-methyl-L or D-tryptophan, brasinin and its derivatives (Navoximod (NLG-919), Indoximod, BMS-986205 and Epacadostat (INCB024360) have been investigated in various studies^12–14^. Epacadostat (EPA / INCB24360) is a reversible inhibitor of indole amine 2,3-dioxygenase-1 (IDO-1) which inhibits IDO-1 by competitive inhibition without interfering with IDO-2 or TDO^15,16^. The clinical effects of this compound have been demonstrated by injecting two subcutaneous doses in a B16 melanoma mouse model. Numerous studies have shown the effectiveness of Epacadostat in the treatment of patients with advanced melanoma^17^.

Despite the great potential of tumor vaccines, conventional vaccination approaches have failed to induce successful treatment and eradication of tumors due to insufficient induction of immune responses^18^. Development of new methods for transferring tumor antigens and inducing an effective immune response is of highly importance^19,20^.

Nanoparticles improve the effectiveness of cancer vaccines by facilitating antigen delivery and T cell activation^21^. This is achieved by efficient transfer of nanoparticles to lymphoid tissues and their prolonged persistence in these tissues as well as controlled release of antigens and adjuvants^22^. Liposomes are bilayer lipid membranes with an inner aqueous chamber^23^. The function of liposomes is highly dependent on their physicochemical properties. In particular, the surface charge has a major effect on their adjuvant properties, and most in vivo studies have shown that cationic liposomes are efficient carriers for anticancer vaccines compared to anionic and neutral liposomes^24,25^. In addition to efficient antigen transport by cationic liposomes, cationic liposomes also show immunogenic properties^26^. Cationic liposomes bind nonspecifically through their positive charge to negatively charged cell membrane of antigen presenting cells like dendritic cells and leads to efficient uptake. Moreover, Cationic liposomes cause depot formation and make efficient uptake by dendritic cells which is an important point in vaccinology^27,28^.

In our previous study, a new liposomal EPA formulation was developed to reduce doses, side effects, and treatment costs compared to the simpler forms of EPA^29^. Co-delivery of Epacadostat with doxorubicin showed that the liposomal system increases the anti-metastatic function in melanoma^30^. This shows the important role of nanoparticle in potentiating cancer immunotherapy by effectively affecting tumor microenvironment by modulating immune cells or immune-influencing factors^31–33^.

Several studies represented the results of combining IDO inhibitors with other immunotherapeutic therapy such as cancer vaccines or cytotoxic chemotherapy. Pre-clinical model of breast cancer demonstrated the synergistic effect of Indoximod and Paclitaxel combination therapy resulted in tumor regression^34^. IDO inhibitors were also used as a combination therapy with sipuleucel-T, or adenovirus-p53 transduced DC vaccine^35,36^.

Several tumor-related antigens including gp100 are overexpressed in advanced melanoma^37^. Multiple studies have shown that the liposomal form of gp100 peptide is able to activate antigen-specific protective immune responses in the melanoma mouse model compared to its simple forms^38,39^.

In the previous study of our group, simple liposomal formulation of DOTAP:Chol was used as a delivery vehicle. In this study, we used the gp100 liposomal vaccine with different formulations, and we developed a new formulation by adding dioleoylphosphatidylethanolamine (DOPE) for encapsulation of gp100 into liposomes which showed a significantly higher encapsulation rate compared to other formulations^40^.

**Figure 1:**
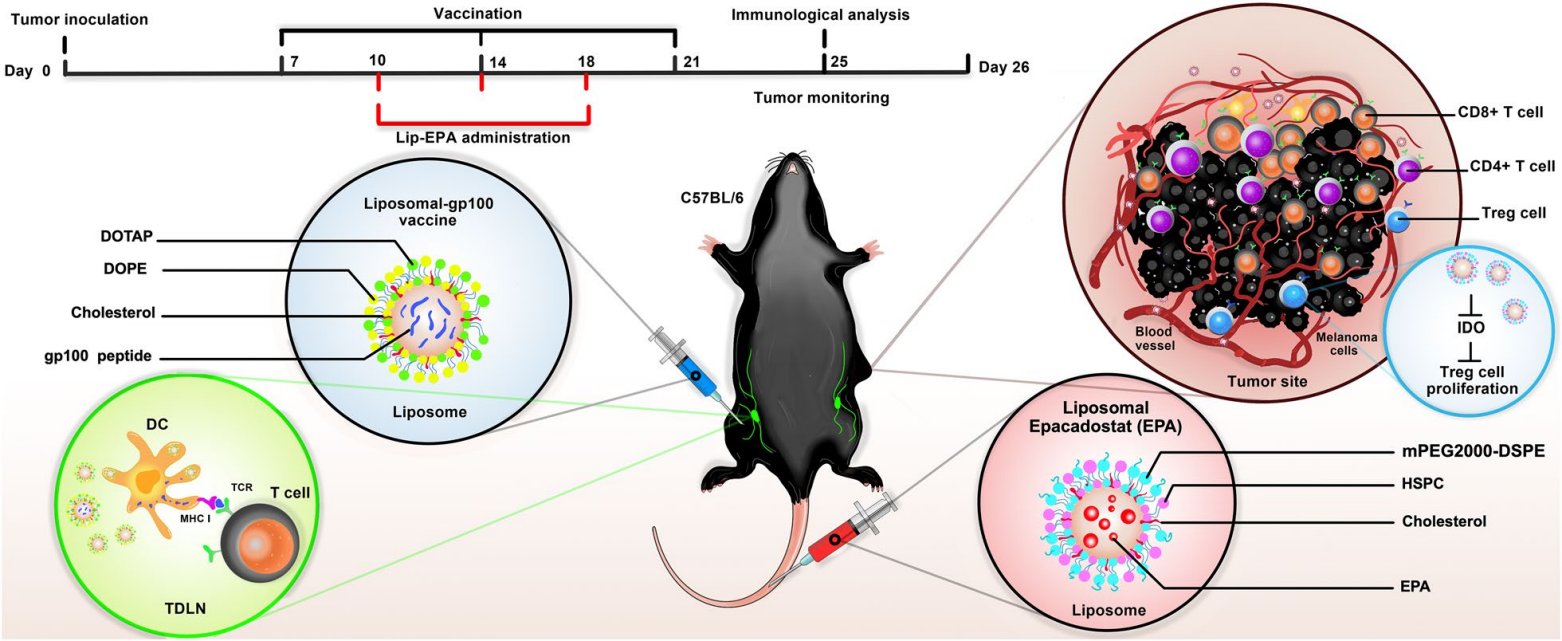
Schematic illustrations of liposomal gp100 formulation used as a therapeutic vaccine in combination with liposomal Epacadostat as an IDO inhibitor in B16F10 melanoma mouse model.

The aim of the present study was to evaluate the effect of a new formulation of gp100 vaccine encapsulated in liposomes in combination with liposomal Epacadostat in a melanoma mouse model. To this end, we combined two distinct strategies in the treatment of cancer, including the use of an IDO inhibitor and a gp100 vaccine encapsulated in liposomes (Fig. 1). The use of EPA as an IDO inhibitor simultaneously with the liposomal peptide vaccine, decreased tumor microenvironment tolerance and increased the immune response against tumor correspondingly.

## Materials and methods

### Materials

EPA was purchased from MetonChem (China). Hydrogenated soy phosphatidylcholine (HSPC), cholesterol (Chol), mPEG2000-distearoylphosphatidyl-ethanolamine (mPEG2000-DSPE), N-[1-(2, 3-Dioleoyloxy) propyl]-N, N, N-trimethylammoniummethyl-sulfate (DOTAP) and dioleoylphosphatidylethanolamine (DOPE) were purchased from Avanti Polar Lipids (Alabaster, AL, USA). Hyaluronidase enzyme was purchased from Sigma-Aldrich (Steinheim, Germany). Collagenase Type I enzyme was purchased from Gibco (UK). Phytohemagglutinin (PHA), Calcein AM (AM = acetoxymethyl) were purchased from Invitrogen (Carlsbad, CA). All flow cytometry antibodies and kits were purchased from BD Biosciences (SanDiego, USA). All solvents and materials were purchased from Merck (Germany) and were in the analytical grade.

### Animals

C57BL/6 mice, 6–8 weeks old, were obtained from the Pasteur Institute (Tehran, Iran). Mice were kept on a 12 h light/12 h dark cycle at a temperature of 22–25 °C. All mice were maintained under pathogen-free conditions with water, and food was given ad libitum. All experimental protocols were approved by Institutional Reseach Ethics Committee, School of Pharmacy, Mashhad University of Medical Sciences (Majid Ghayour-Mobarhan, Mohammad Ali Kiani, Bibi Sedigheh Fazli Bazzaz, Mehrdad Iranshahi) and all methods were carried out in accordance with the guidlines issued by Research Advisory of Mashhad University of Medical Sciences (Grant number: 970822).This study was carried out in compliance with the ARRIVE guidelines.

### Cell line and media

B16F10 cells, a metastatic melanoma cell line expressing gp100 antigen, were obtained from the nanotechnology department of Bu-Ali Research Institute of Mashhad. The cells were cultured in DMEM medium containing 10% FBS and 1% penicillin–streptomycin at 37 °C and 5% CO_2_. The CT26, murine colon carcinoma cell line, was purchased from Pasteur Institute (Tehran, Iran) and cultured in RPMI-1640 medium supplemented with 10% FBS.

### Peptide

The peptide used in this study was modified gp100, with a molecular weight of 1114.19 D and purity of 99.33%, which consisted of 9 amino acids and contained sequences Glu-Gly-Pro-Arg-Asn-Gln-Asp-Trp-Leu. The peptide was designed by ChinaPeptides (Shanghai, China) and purchased as a lyophilized powder. The powder, approximately 15 mg, was dissolved in 15 ml of solution (99.99% H_2_O / 0.1% NH_4_OH) to obtain a concentration of 1 mg /ml.

### Selection of the optimum formulation for melanoma peptide vaccine (gp100)

#### Preparation of liposomes

Liposomal formulation was prepared based on our previous study^41^. Briefly, a 12 mM liposomes containing DOTAP: DOPE : Chol lipids with a molar ratio of 4: 4: 4 was prepared. The required volume of the stock solution of 40 mg/ml of each lipids in chloroform was placed in a rotary vacuum for 2 h at a temperature of 40–45 °C. Then, it was placed in the freeze-dryer for 2 h to completely remove the residual chloroform and stored in − 20 °C under Argon gas until to be used.

#### Peptide loading into liposomes

Peptide-loaded liposomes prepared based on thin film dispersion-extrusion method. Briefly, the lipid film was hydrated with sterile distilled water at 45 °C. Simultaneously, the peptide with a concentration of 100 μg/ml was added and mixed by vortexing for 30 min at 45 °C. The obtained milky solution containing multilamellar vesicles (MLVs) was sonicated at 45 °C for two minutes. The solution was then filtered 15 times through 200 nm and 100 nm filters using an extruder (Avestin, Canada). The prepared liposome was transferred into a dialysis tube (10–12 kD) and dialysis was performed three times using a 10 mM HEPES buffer (pH 7.4) containing 9.5% w/v of sucrose, for 2, 4, and 12 h.

### Determining of particle size and peptide encapsulation efficiency of liposomal gp100

The Particle Size Analyzer and Zeta Sizer (Nano-ZS; Malvern, UK) was used to evaluate the size and zeta potential of-liposomal formulations. The percentage of encapsulation efficacy (EE%) of each formulation was determined by a high performance liquid chromatography (HPLC) method. The UV detector was set at a wavelength of 220 nm. Different concentrations of peptide including 6.25, 12.5, 25, 50, and 100 µg/ml were prepared from a stock peptide solution and injected in triplicates into the device to obtain the calibration curve.

The empty liposomes were dissolved in an acidic isopropyl alcohol and injected into the machine as a control. The results of the set-up were analyzed as area under the curve (AUC) and the loading percent was obtained. Peptide concentration in each formulation was measured before and after dialysis to determine the encapsulation efficiency.

### Evaluation of IDO expression in B16F10 melanoma cells using qualitative PCR

B16F10 cell line was cultured in DMEM medium containing 10% v/v FBS and 1% w/v penicillin–streptomycin. After sufficient proliferation, cells were trypsinized and prepared for injection into female C57BL6 mice (n = 5). A total of 5 × 10^5^ cells in 60 μl PBS were injected subcutaneously into the right side of each mouse. Fourteen days after injection, mice were sacrified by cervical dislocation. Tumor tissues were examined to assess the expression of IDO using conventional PCR technique. RNA was extracted from the target tissues using an RNA isolation kit (DENA ZIST ASIA) and the concentration of RNA was measured using nanodrop at 260 and 280 nm wavelengths. The extracted RNAs were converted to cDNA using cDNA Synthesis kit (YektaTajhizAzma). IDO specific primers were designed and synthesized by the German company Metabion. We used Taq DNA polymerase enzyme. Thermal cycling conditions used were: 95 °C for 5 min followed by 40 cycles at 60 °C for 1 min, 72 °C for 30 s and 45 °C for 30 s with total run time of approximately 2 h. Qualitative expression of the PCR product (123 bp) was assessed by 2% agarose gel electrophoresis.

### In vivo evaluation of the toxicity of Epacadostat by MTD method

Six C57BL/6 mice, aged 6–8 weeks, were selected for this experiment. Their initial weight was taken just before the experiment. Three different doses of EPA including the highest dose (7.5 mg/kg), the average dose (3.5 mg/kg) and the lowest dose (1.5 mg/kg) were used via the intravenous injection into the tail vein of mice (two mice for each doses). The mice were then monitored for two weeks. Mice were sacrified when the weight loss reached more than 15% of the initial weight.

### Study of drug biodistribution in the tumor tissue

#### Preparation and characterization of liposomal Epacadostat containing indocyanine green (ICG) dye

Indocyanine green (ICG) dye was used to label liposomes. Briefly, 1.1 mg of ICG dye was added to the lipid mixture (HSPC: Chol: mPEG-DSPE lipids at concentrations of 57: 38: 5 molar ratio) into round bottom glass flasks during preparation process. Then, liposomes containing EPA were formulated using remote loading method according to our previously published protocol^29^. According to the physicochemical characteristics of Epacadostat which is the hydrophobic (PKa = 4) and also the IC_50_, we use 200 µgof the drug for loading in the liposome. Briefly, 200 µg of EPA was added to 1000 µl of ICG-labeled empty liposome and placed in a 70 °C water bath in for 20 min. Then, the formulation was immediately transferred to the cold-water bath to reach a temperature below Tm of the liposomes and dialyzed in the presence of 10 mM histidine buffer containing 10% w/v of sucrose (pH 6.8). The encapsulation efficacy of both ICG and EPA was calculated by dissolving liposome bilayer in methanol and stirred in a water bath at 60◦C for a few minutes. Then the absorption of empty liposome and lysed sample were read using a spectrophotometer at 287 nm and the percentage of encapsulation was calculated by the following formula:

$$\mathrm{\% EE}= \frac{the \, amount \, of \, Epacadostat \, in \, the \, formulation}{the \, added \, amount \, of \, Epacadostat} \, \times \, 100.$$

### Scintigraphic imaging

Three C57BL/6 mice were weighted at the beginning of the study. After anesthesia, 5 × 10^5^ cells in 60 µl PBS were injected subcutaneously (s.c.) into animals. Fourteen days after tumor injection, a safe dose of ICG-labeled Lip-EPA was injected through the lateral tail vein, based on toxicity and maximum tolerated dose. Mice were anesthetized and fixed, and planar scintigraphic imaging was performed at 0, 3, 24, and 48 h post-administration using the imaging technology, KODAK In Vivo Imaging System F Pro (Eastman Kodak Company; molecular imaging system; USA, Excitation: 760 nm, Emission: 860 nm).

### In-vivo evaluation of antitumor efficacy of the combination therapy

#### Tumor induction and treatment schedule

After proliferation of B16F10 cells, 5 × 10^5^ log phase cells in 60 μl were subcutaneously injected into 70 female C57BL/6 mice weighting 18 ± 3 g. Seven days after tumor inoculation, tumors were observed (~ 5 mm in length or width) in mice and then, mice were randomly divided into 7 groups, 10 mice each, including group (1) PBS as a control, group (2) gp100, group (3) Epacadostat, group (4) Lip-gp100, group (5) Lip-Epacadostat, group (6) Epacadostat + gp100 and group, (7) Lip-Epacadostat + Lip-gp100. The mice were treated with three intravenous doses of Epacadostat (60 µg/mouse) at four-day intervals started on day 10 and three subcutaneous (in the groin) doses of gp100 melanoma vaccine (25 µg/mouse) at seven-day intervals started on day 7.

### Evaluation of tumor size and survival rate

Four days after the last treatment injection, 3 mice from each group were sacrified and subjected to in vitro testing. The other 7 mice were monitored for tumor size and survival rate three times a week until 28 day after tumor injection. Tumor size was measured using caliper and calculated using the formula V = 0.5 × (Length × width^˄2^). The time to reach the end point (TTE) criteria of mice were as follows: (a) body weight dropped below 15% of their initial weight, (b) the tumor masses greater than1000 mm^3^ and (c) declining health or obvious signs of sickness and lethargic. Kaplan–Meier estimator test was used to determine the survival rate of mice. Moreover, we calculated TTE, tumor growth delay (%TGD), median survival time (MST), and increased life span (% ILS) for each group^42^. Time to reach the end-point (TTE) for each mouse was calculated based on the equation of the line obtained by exponential regression of the tumor growth curve.

### Single cell preparation

Four days after the last treatment, 3 mice from each group were sacrified and their spleen, inguinal lymph node, and tumor tissue were dissected and mashed through a 70-µm cell strainer. Tumor tissues were digested primarily by incubation with Collagenase type I and Hyaluronidase for 2 h at 37 °C. The RBCs were depleted using ACK lysing buffer. Then the isolated single cells from spleens and lymph nodes and cells in tumors were used for immunological analysis.

### In vitro evaluation of antitumor efficacy of the combination therapy

#### Evaluation of lymphocytes population in the spleen, lymph node and tumor infiltrated cells using flow cytometric analysis

Isolated cells (1 × 10^6^ cells) from each tissue were seeded in 24-well plates, stimulated with 10 µg/ml peptide and incubated at 37 °C for 18 h. 10 µg /ml of brefeldin A solution was added to each well and placed at 37 °C for 6 h. As a positive control of each group, 10^5^ cells /ml was seeded onto 10 wells of a 24-well plate treated with 10 µg /ml cell activation cocktail with brefeldin A for 6 h. After incubation time, 2 × 10^5^ cells were added to flow cytometry tubes and anti- CD3, CD4, CD8, CD25, FOXP_3_ antibodies were used to determine the number of CD4^+^ T cells, CD8^+^ T cells, and Treg cells. IFN-γ, IL-4 and IL-10 secretion were also assessed by intercellular staining using 1X fix/perm. The data were then acquired using a flow cytometer (BD FACSCalibur, BD Biosciences) and analyzed using FlowJo software (v7.6.1).

#### Evaluation of IFN-γ secretion by Enzyme-linked immunospot (ELISpot) assay

In order to evaluate the activity of immune cells in treated mice, the secretion of interferon gamma on stimulation with gp100 peptide was examined by ELISpot assay. For this purpose, the activity of lymphocytes in terms of interferon gamma secretion in spleen, lymph node, and tumor tissues was assessed by binding of this cytokine to the antibody coated at the bottom of the plate and the formation of spots were investigated after the addition of the usual substrate. The experiment was carried out according to the manufacturer’s protocol (Mouse IFN-γ ELISpot BASIC, Mabtech AB, Sweden). Spots were counted with the aid of an image analyzer software (version 3.5, Eastman Kodak, Rochester, New York).

### In vitro cytotoxicity assay

In order to access the cytotoxic activity of CTLs, splenocytes (as effector cells) isolated from treated mice (three per group) were co-cultured at different ratios with Caleine AM-labelled B16F10 cells (2 × 10^4^, as target cells) in U-bottomed plates in triplicate wells and incubated for four hours at 37 °C. CT26 cell line which does not express gp100 antigen was also labelled with 12.5 μM Calceine AM at 37 °C for one hour in the dark and used as negative control^43^. Culture medium containing 2% Triton X-100 and without Triton X-100 solution were used to determine the maximum and minimum release by target cells, respectively. Fluorescence of Calein AM released in supernatants was read on a luorimeter (FLx800, BioTek Instruments Inc. USA) with excitation at 485 nm and emission at 538 nm. The specific lysis was calculated as follows: percentage of specific lysis = (release by CTLs − minimum release by targets)/(maximum release by targets − minimum release by targets).

### Evaluation of IDO expression in tumor tissue of treated mice using Real Time PCR technique

Four days after the last injection, 3 mice from each group were sacrified and their tumor tissues were isolated. RNA isolation kit (DENA Zist Asia, Iran) was used to extract RNA from tumor tissues and cDNA was synthesized according to the manufacturer’s instruction (Yekta Tajhiz Azma, Iran). Real-time PCR (SYBR-Green) was used to assess quantitative expression of mouse IDO gene by IDO specific primers and GAPDH internal primer using Rotor-Gene Q instrument. Specifications and sequences of mouse GAPDH and IDO-specific primers are listed in Table 1. In summary, 10 µl mastermix, 1 µl forward primer, 1 µl reverse primer, 6 µl distilled water and 2 µl cDNA were mixed in a final volume of 20 µl. The results were calculated as dCt (Ct (IDO)—Ct (GAPDH) followed by ddCt for IDO expression level in each treatment groups relative to control group (dCt (treatment groups)—dCt (control group)) and finally presented as a fold change (2^(-ddCt)).

**Table 1:** Specifications and sequences of IDO and GAPDH specific primers.

Target genes	Sequences of primers	Template length (bp)	Melting Temperature (°C)
IDO	*F-5*′ GCCTGCCTCCTATTCTGTCT*3*'	120 bp	82 °C
	*R-5*′ GAAGCTGCGATTTCCACCAA*3*'	120 bp	82 °C
GAPDH	*5*′ TGCACCACCAACTGCTTAG *3*'	80 bp	81 °C
	*5*′ GATGCAGGGATGATGTTC *3*'	80 bp	81 °C

### Statistical analysis

Normal distributed quantitative variables were analyzed using one-way and two-way analysis of variance (ANOVA) followed by Tukey post hoc multiple comparisons test and were expressed as mean ± SD. Survival data was analyzed by the Kaplan–Meier log-rank (Mantelcox) test. Qualitative variables were analyzed using χ^2^ test. A probability level of less than 0.05 was considered as being significant.

## Results

### Encapsulating of the gp100 peptide and evaluation of its physicochemical properties

Encapsulation of the gp100 vaccine and its physicochemical properties were shown in the Table 2. The liposome formula showed a size of 154 nm and a positive charge. PDI level was acceptable, reported less than 0.1 which indicates the homogenicity of liposome size. The percentage of encapsulation efficacy of the peptide was 59%.

**Table 2:** The physicochemical properties of liposomal formulation and percentage of encapsulation efficacy.

Formulation	Liposomal composition	Molar ratio	Size number	Z average^a^	PDI^b^	Zeta potential	Encapsulation efficacy %
FL1	DOTAP:DOPE:Chol:gp100	4:4:4:100	100	154 ± 4	0.01 ± 0.00	12.5 ± 0.1	59.1 ± 1.0

(mean ± SD; n = 3).

### The IDO expression in the B16F10 melanoma tumor

**Figure 2:**
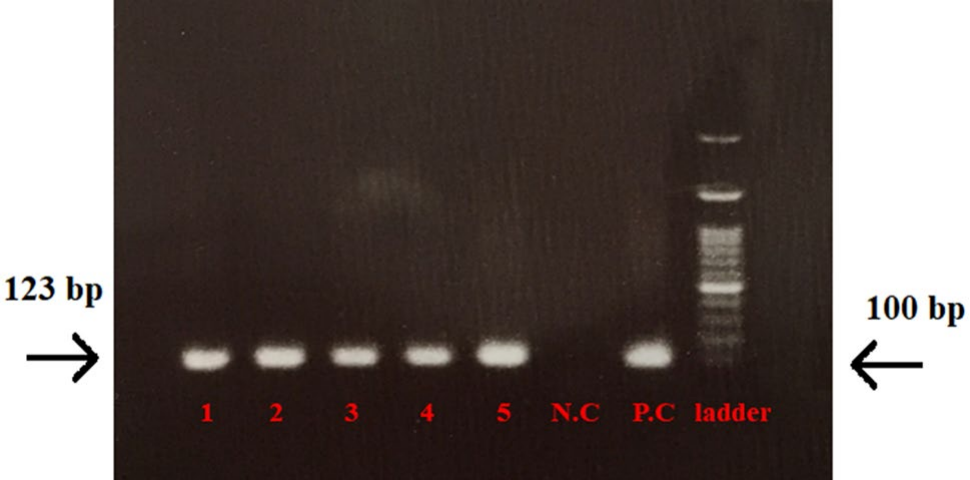
Agarose gel electrophoresis of the PCR product of B16F10 tumor. Purification of amplified IDO fragment. Lanes 1 to 5: B16F10 tumor samples, *NC* blank control without template (negative control), *PC* 4T1 cell line (positive control) single 123 bp band corresponded to IDO gene, Ladder: 100 bp DNA marker.

After induction of the tumor in the C57BL/6 mice, the tumor tissue was taken out through surgery, and its RNA was extracted. Afterwards, IDO’s qualitative expression was evaluated by the PCR test. The results showed that IDO was expressed in all of the B16F10 (n = 5) tumor bearing mice (Fig. 2). The PCR product lengths was in the limit of $200bp$.

### Determination of MTD of Epacadostat

After injecting various doses ($1.5 , 3.5 ,and 7.5 mg/kg$) into the mice and evaluation of the weight loss and casualties, maximum tolerated dose was obtained by $3.5 mg/kg$. Mice with the injected dose $7.5 mg/kg$ lost their weight (less than 15% of their initial body weights) as well as having casualties during a three-week investigation (Fig. S1).

### The physicochemical properties of ICG-labeled Lip-EPA

The particle size, zeta potential, and encapsulation efficacy of ICG-labeled Lip-EPA and Lip-EPA is presented in Table 3. The amount of ICG dye was chosen according to the previous studies^44–46^. The results showed that labeling had no negative effect on encapsulation efficacy of EPA. On the other hand, the zeta potential became a little bit negative after Lip-EPA labeling. PDI level for the ICG-labeled Lip-EPA was acceptable, reported less than 0.1. But the size of ICG- labeled Lip-EPA was slightly larger than Lip-EPA.

**Table 3:** The physicochemical properties of liposomal formulations of Lip-EPA and ICG-labeled Lip-EPA and percentage of encapsulation efficacy.

Formulations	Composition	Molar ratio	Total lipid concentration mM	Z-average^a^ (nm) ± SD	PdI^b^	Z-potential (mV) ± SD	EE%
Lip-EPA	HSPC:Chol:MPEG-2000-DSPE:EPA	57:38:5:200	50	128.1 ± 1.1	0.09 ± 0.0	− 16.5 ± 1	64.9 ± 3.5
ICG-labeled lip-EPA	HSPC:Chol:MPEG-2000-DSPE:EPA:ICG	57:38:5:200:1100	50	178.3 ± 0.1	0.09 ± 0.0	− 17.9 ± 1	63.6 ± 1.7

(mean ± SD; n = 3).

### Accumulation of ICG-labeled Lip-EPA in the tumor tissue

**Figure 3:**
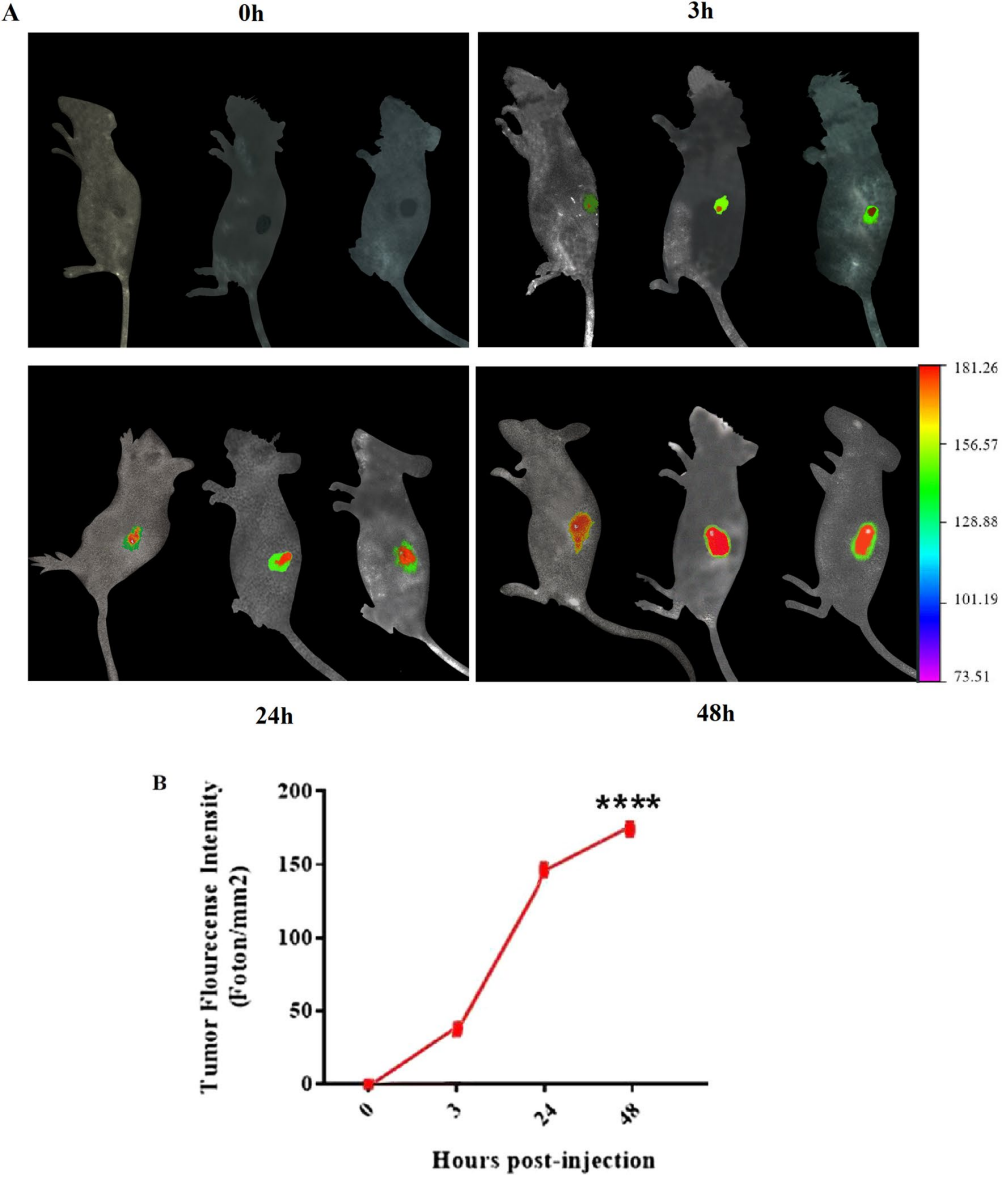
In vivo fluorescence imaging and the associated fluorescent intensity of tumor in C57BL/6 tumor bearing mice. (**A**) Representative whole-body images of ICG-labeled liposomal-EPA at different time points post *i*.*v*. administration via lateral tail vein. Fluorescence intensity scale is displayed on the right side of the images. (**B**) Associated fluorescence intensity in tumor site at different time points (n = 3).

The drug was injected intravenously and the mice were photographed at different time points. As shown in Fig. 3A, accumulation of the drug in the tumor was observed at 3 h after injection. The increase ofliposomal drug accumulation in tumor tissue at 24 h after injection was greater than in the tumor at 3 h. At 48 h after injection of the labeled liposome, the highest accumulation was observed in the tumor area. The results showed that there was a significant (p < 0.0001) difference in the fluorescence intensity observed in the tumor tissue at different time points after injection (Fig. 3B).

### In vitro efficacy of the combination therapy in the tumor bearing mice

#### The frequency of T cell population and intracellular cytokine analysis

Four days after the last therapeutic injection, 3 mice per each group were sacrificed, and their spleen, lymph node, and tumor tissue were detached. The phenotype of cells and their secreted cytokines were assessed using the flow cytometry technique.

**Figure 4:**
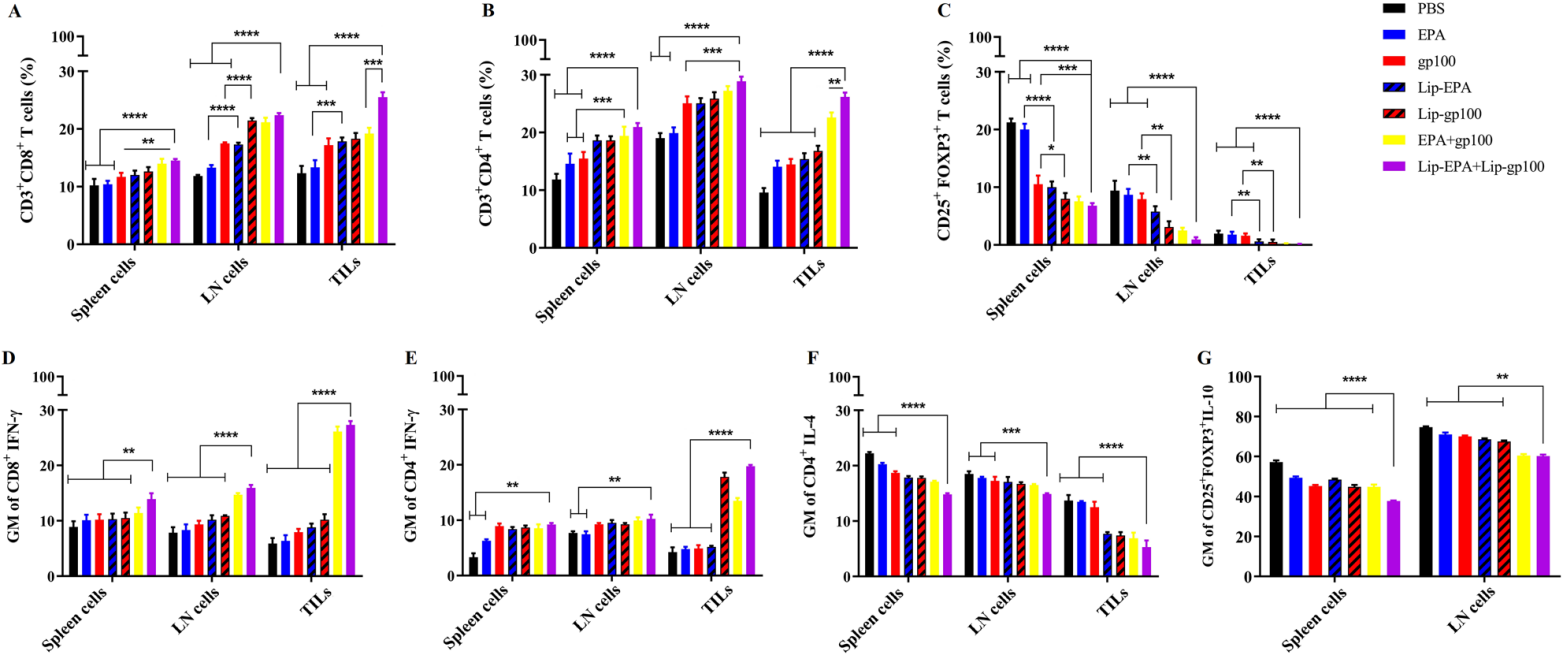
The Flow cytometric analysis of lymphocyte populations in spleen, lymph node, and the tumor site. (**A**) The frequency of CD3 + CD8 + T cells, (**B**) CD3 + CD4 + T cells and (**C**) CD25 + FOXP3 + Treg cells in different tissues from mice received different treatments. The geometric mean of IFN-γ- producing splenic/ lymph node/ intra tumoral (**D**) CD3 + CD8 + T cells, (**E**) CD3 + CD4 + T cells. (**F**) The geometric mean of IL-4-producing splenic/lymph node/intra tumoral CD3 + CD4 + T cells and (**G**) The geometric mean of IL-10-producing splenic/lymph node CD4 + CD25 + FOXP3 + Treg cells. Data are represented as mean ± standard deviation (n = 3). *p < 0.05, **p < 0.01, ***p < 0.001, **** p < 0.0001. *LN* lymph nodes, *TILs* tumor infiltrated lymphocytes.

Based on gating strategies (Fig. S2) and according to statistical data analysis, a considerable difference in the population of the CD8^+^ T cells of the spleen tissue was spotted among various treatment groups. A significant difference in CD8^+^ T cells was found between the mice received EPA (p < 0.0001), or gp100 (p < 0.01) monotherapy and those recieved the combination therapy (i.e. Lip-EPA + Lip-gp100). Analysis of the lymph node tissue was demonstrated that the liposomal combination therapy group was of the greatest number of the lymphatic tissue CD8^+^ T cells relative to the other groups. Data of the tumor tissue also demonstrated the same trend of the liposomal combination therapy group compared to the other groups.The liposomal formulation also was superior (p < 0.001) than non-liposomal form of EPA + gp100 (Fig. 4A).

Scrutiny of the spleen tissue demonstrated that a significant difference in the populations of CD4 + T cells was found between the mice received EPA or gp100 monotherapy groups’s and those which recieved the EPA + gp100 or Lip-EPA + Lip-gp100 combination therapy (p < 0.001 and p < 0.0001, respectively). The significant difference was also found between the population of the monotherapy groups in lymph node CD4 + T cells and that of the combination therapy group Lip-EPA + Lip-gp100 (p < 0.0001 and p < 0.001, respectively). The tumor tissue alaysis demonstrated the greatest differences relative to two other tissues in the liposomal combination therapy group (Lip-EPA + Lip-gp100) in the number of the CD4 + T cells over monotherapy and non-liposomal EPA + gp100 groups (Fig. 4B).

Analysis of the spleen tissue demonstrated that a significant difference was found in the population of CD4 + CD25 + FOXP3 + T cells in the EPA monotherapy group compared to the combination therapy groups EPA + gp100 or Lip-EPA + Lip-gp100 (p < 0.0001). The significant difference in the number of CD4 + CD25 + FOXP3 + Tcells in the lymph node and tumor tissues was found in the population of the EPA and gp100 monotherapy groups compared to the combination therapy groups EPA + gp100 and Lip-EPA + Lip-gp100 (p < 0.0001) (Fig. 4C).

In the intracellular cytokine analysis of T cell populations, it was found that the geometric mean of IFN-γ- producing CD3^+^CD8^+^ T cells in the spleen, lymph node, and tumor site in the mice received Lip-EPA + Lip-gp100 combination therapy was more than that of the other groups (p < 0.01 in spleen, p < 0.0001 in lumph node and tumor site; Fig. 4D).

The same results were demonstrated for geometric mean of IFN-γ- producing splenic/lymph node/intratumoral CD3^+^CD4^+^ T cells in the Lip-EPA + Lip-gp100 combination therapy group compared to other groups with significantly higher difference in tumor site (p < 0.0001) relative to spleen and lymp node tissues (p < 0.01) (Fig. 4E). On the other hand, the geometric mean of IL-4- producing splenic/lymph node/intratumoral CD3^+^CD4^+^ T cells in the Lip-EPA + Lip-gp100 combination therapy group was less than those of the other groups (p < 0.0001) (Fig. 4F).

The geometric mean of IL-10- producing splenic/lymph node CD4^+^FOXP3^+^ T cells in the Lip-EPA + Lip-gp100 combination therapy group was less than those of the other groups (p < 0.0001 and p < 0.01, respectively) (Fig. 4G).

### The evaluation of the IFN-γ production in treated mice

**Figure 5:**
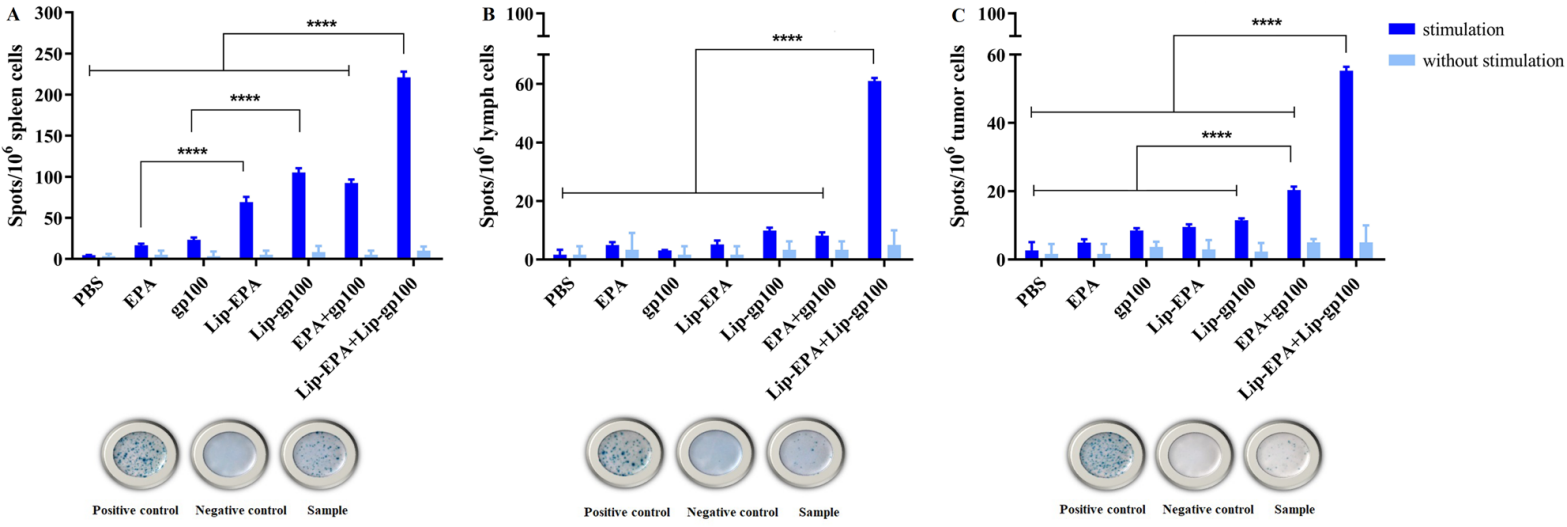
Interferon (IFN)-γ-secreting cells in spleen, lymph node and tumor tissues of the different treatment groups. IFN-γ-producing cells in (**A**) spleen, (**B**) lymph node and (**C**) tumor site as spot-forming cells (SFCs)/1000,000 cells. Data are represented as mean ± standard deviation (n = 3). ****p < 0.0001.

The results of ELISpot test were calculated based on the average Spot ± SD in three wells performed in each group and finally reported as SFC (Spot-forming cells) per 10^6^ cells. According to two-way ANOVA statistical test and data analysis, a significant difference was observed in terms of IFN-γ production between the combination therapy group Lip-EPA + Lip-gp100 and the other groups in spleen (Fig. 5A) and lymph node (p < 0.0001) (Fig. 5B). In the results of the tumor tissue analysis, besides the significant difference in Lip-EPA + Lip-gp100 group, this difference was also shown between EPA + gp100 group and other groups ( p < 0.0001) (Fig. 5C).

### The cytotoxic activity of CTL cells of treated mice

**Figure 6:**
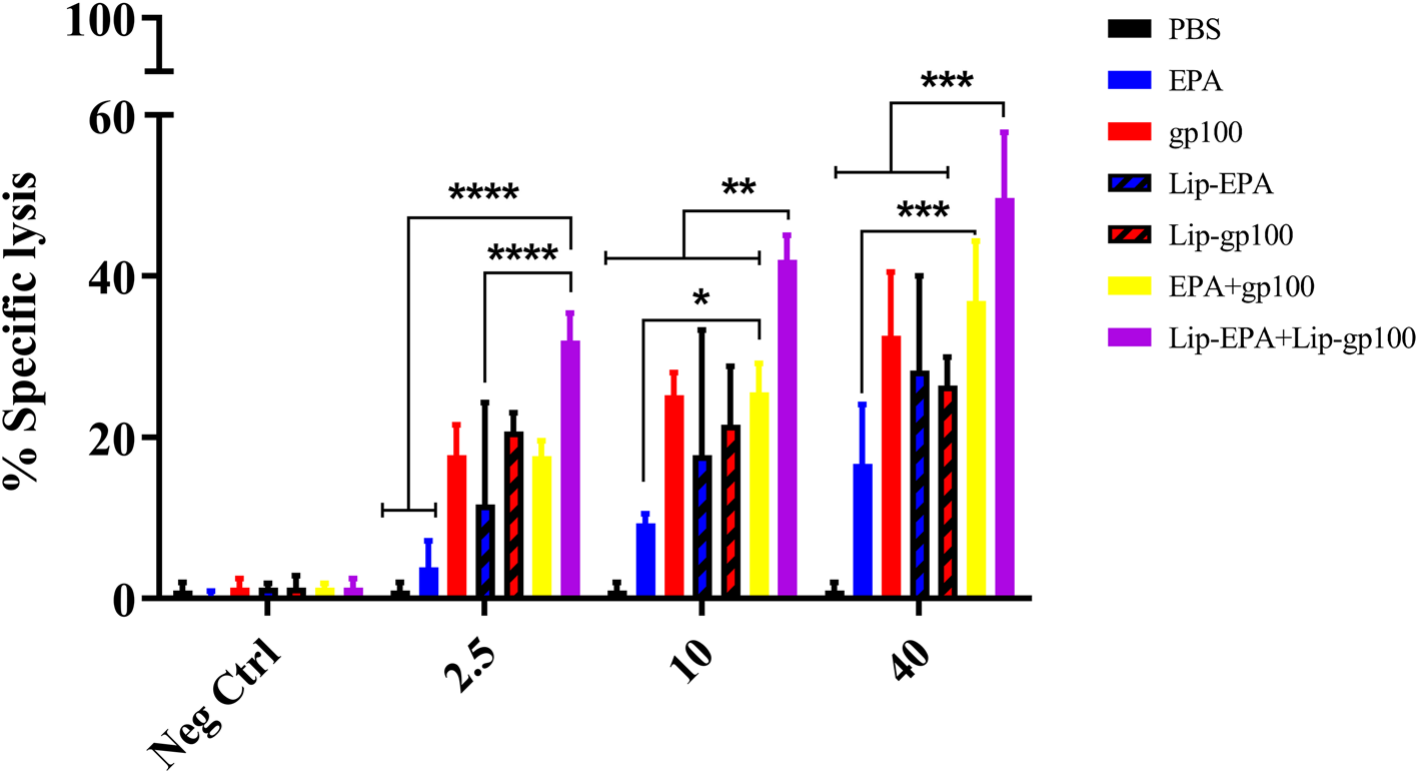
The cytotoxic activity of CTL cells. Antigen-specific cytotoxic activity of CTLs was assessed using different ratios of effector (splenocytes) to target (Calcein AM-labelled B16F10 cells expressing gp100 antigen) cells (E/T). Calcein AM-labelled CT26 cell line was used as negative control. Data are represented as mean ± standard deviation (n = 3). *p < 0.05, **p < 0.01, ***p < 0.001, ****p < 0.0001.

In vitro analysis of CTLs activity showed that in the ratio of 2.5/1, there was a significant difference between the cytotoxic activity of CTL cells in the combination therapy group Lip-EPA + Lip-gp100 and the PBS, EPA or EPA-Lip (p < 0.0001). In the ration of 10/1, the Lip-EPA + Lip-gp100 again had significant difference over other groups (p < 0.01). In this ratio the EPA + gp100 formulation also showed significant difference over EPA (p < 0.05). At a dilution of 40/1, a significant difference was found between the combination therapy group Lip-EPA + Lip-gp100 and the other groups except the EPA + gp100 group (p < 0.001) (Fig. 6).

### Evaluation of IDO-1 expression in the tumor tissue of treated mice

**Figure 7:**
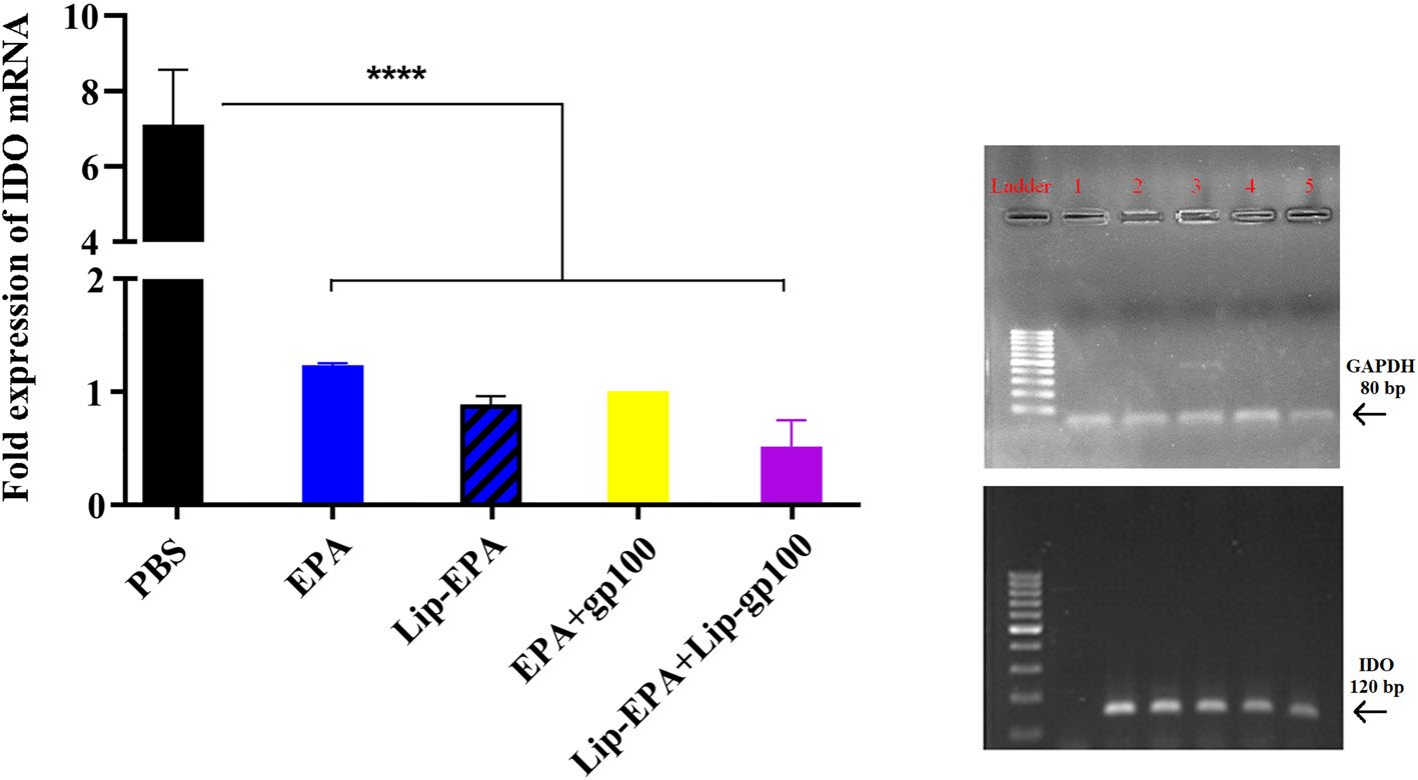
The relative changes in mRNA expression level of the IDO gene in the different treatment groups. Real-time quantitative PCR was used to quantify IDO mRNA expression level in tumor site relative to housekeeping gene, GAPDH. Quantitative results are expressed as the mean ± standard deviation (n = 3). ****p < 0.0001.

The mean ∆ct was calculated for the IDO and GAPDH genes. The results showed that the mRNA expression of the IDO gene was decreased significantly in all treated groups compared to the PBS control group (p < 0.001) (Fig. 7). But, there was no significant differencs in mice received combination therapy in liposomal or free form.

### In vivo efficacy of combination therapy in tumor bearing mice

#### Evaluation of the tumor growth

**Figure 8:**
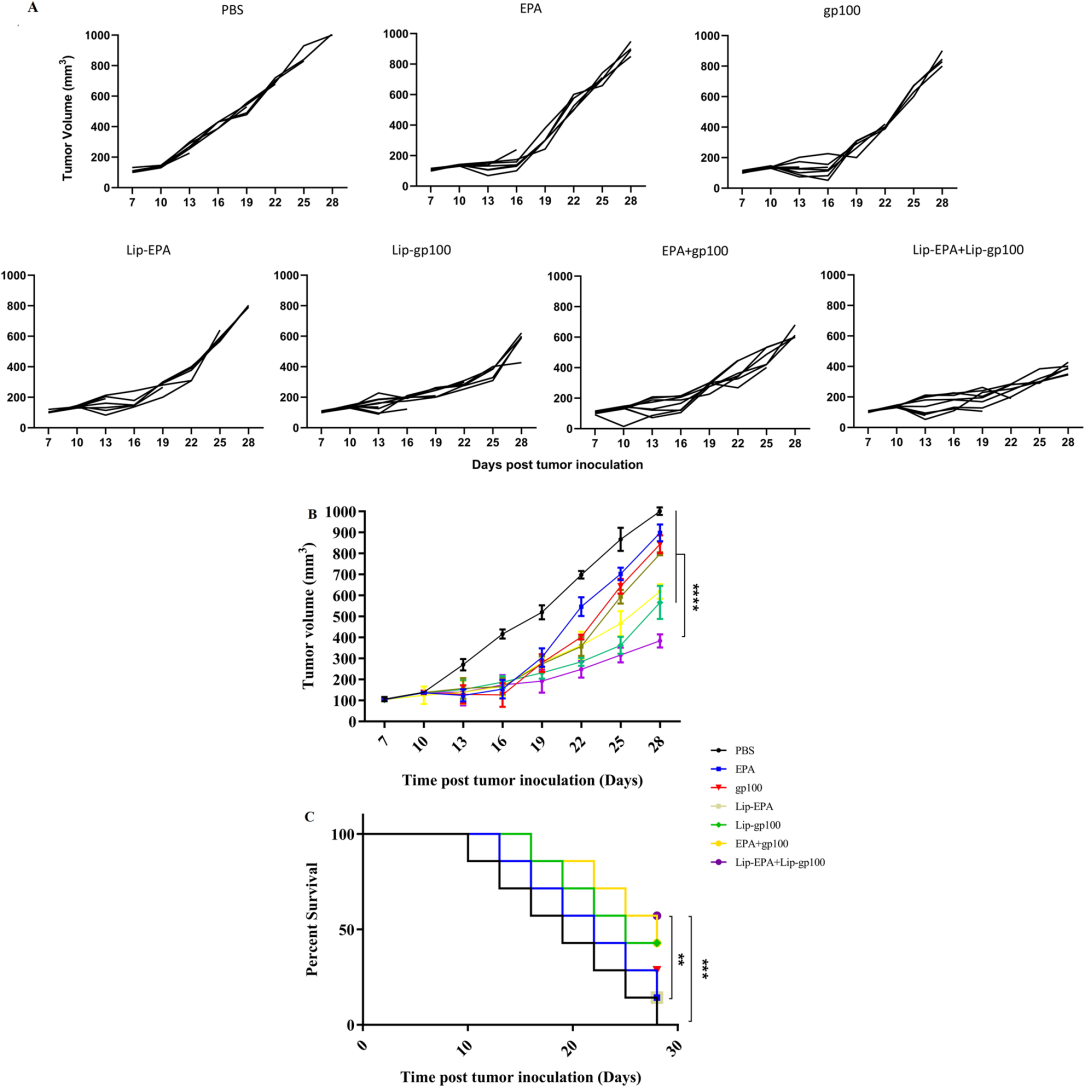
Kinetics of tumor growth and survival analysis of B16F10 melanoma tumor bearing mice treated with a combination of liposomal gp100 anticancer vaccine with liposomal EPA as a IDO inhibitor. (**A**) Individual tumor growth curves of animals in each group (**B**) Mice received three doses of vaccine and three doses of drug. Tumor size measurements started on day 10 till day 28, three times per week, and presented as tumor volume (mm^3^). Data are represented as mean ± standard deviation (n = 9). (**C**) The survival of mice was analysis using the multiple comparison log-rank (Mantel–Cox) test. *p < 0.05, **p < 0.01, ***p < 0.001, ****p < 0.0001.

The measurement of the tumor emerged in the mice was analysed using the caliper tool three times a week up to 28 days after tumor inoculation. Tumors appeared on day 6 or 7 after challenge with B16F10 cells and reached the size of 5 mm in one side. Tumor size for each mouse group was calculated as the averages of mice’s tumor volumes in that group. Mice treated with Lip-EPA + Lip-gp100 had slowest tumor growth rate and the tumors reached their maximum volumes around 400 mm^3^ 28 days post-tumor inoculation (Fig. 8A). On days 19, 22 and 25 there was a significant difference in the mean tumor volume between the Lip-EPA + Lip-gp100 group and the other groups including non-loposomal EPA + gp100 (p < 0.0001), except for the Lip-gp100 group. On day 28, the last day of monitoring, a significant (p < 0.0001) difference was observed between the combination therapy group Lip-EPA + Lip-gp100 and all other groups even Lip-gp100 group (p < 0.0001) (Fig. 8B).

The results showed that the tumor growth rate was slower significantly in the Lip-EPA + Lip-gp100 group and the mean tumor volume was significantly lower than the other groups. Although the lowest tumor size was found in the Lip-EPA + Lip-gp100 combination therapy group, the tumor size was significantly lower in the Lip-gp100 therapy group than that in the other groups. Also, there was a difference in the mean tumor volume between the EPA and Lip-EPA groups, as well as between the gp100 and Lip-gp100 groups, and the rate of the tumor growth in the Lip-EPA and Lip-gp100 groups was slower than the non-liposomal EPA and gp100 groups (Table S1).

#### Evaluation of the survival rate

Survival rate of the mice was analyzed using GraphPad-Prism-6 software and log-rank mantel test and reported as the percentage. According to Table 4, the lowest incidence of death was related to the liposomal combination therapy group i.e. Lip-EPA + Lip-gp100 . In terms of survival rate, the highest survival was in Lip-EPA + Lip-gp100 group. Log Rank test was significant in terms of the difference between survival rates in different therapy groups. The results of the survival analysis showed the significant difference between the Lip-EPA + Lip-gp100 combination therapy group and EPA, Lip-EPA (p < 0.01), or PBS ( p < 0.001) group over time but there was no significant between Lip-gp100 and Lip-EPA + Lip-gp100 groups. (Fig. 8C).

**Table 4:** Incidence of death and survival rate in different treatment groups.

Groups	Total number of mice	Death incidence	Survival rate N (%)
PBS	7	7	0 (0%)
EPA	7	6	1 (14.2%)
gp100	7	5	2 (28.5%)
Lip-EPA	7	6	1 (14.2%)
Lip-gp100	7	4	3 (42.8%)
EPA + gp100	7	4	3 (42.8%)
Lip-EPA + Lip-gp100	7	3	4 (57.1%)
Overall	49	35	14 (28.5%)

The highest time to reach end point (TTE), median survival time (MST), increased life span (ILS), and tumor growth delay (TGD) were observed in the Lip-EPA + Lip-gp100 combination therapy group (MST > 28 days, TTE = 34.99 ± 11.89 days, ILS > 47.36%, TGD = 56.54%), which was higher than the other groups (Table 5).

**Table 5:** Data on the effectiveness of different treatments in B16F10 tumor bearing mice.

Treatment groups	TTE^**a**^ (days ± SD)	TGD^b^ (%)	MST^c^ (days)	ILS^d^ (%)
PBS	22.35 ± 8.24	–	19	–
EPA	24.36 ± 8.15	9.01	22	15.78
gp100	24.04 ± 8.52	7.55	22	15.78
Lip-EPA	25.12 ± 13.12	12.4	22	15.78
Lip-gp100	30.77 ± 8.80	37.64	25	31.57
EPA + gp100	30.62 ± 9.65	36.99	28	47.36
Lip-EPA + Lip-gp100	34.99 ± 11.89	56.54	> 28	> 47.36

^a^Time to reach end point; ^b^Tumor growth delay; ^c^Median survival time; ^d^Increased life span.

In the study of TTE, a significant difference was observed in Lip-EPA + Lip-gp100 over other groups with p < 0.0001 except Lip-gp100 with p < 0.01. Also significant difference in TTE between the non-liposomal gp100 and the liposomal-gp100 groups (p < 0.0001).

In the study of TGD, a significant difference was observed Lip-EPA + Lip-gp100 compared all other groups (p < 0.0001). In the study of ILS, a significant difference was observed between Lip-EPA + Lip-gp100 over other groups with p < 0.0001 except EPA + gp100 with p < 0.01. (Table S2).

## Discussion

The main goal of the present study was to compare a tumor targetted therapy via liposomes in combination with a cancer vaccine with a nanoliposomal form of EPA and to improve the efficacy of the drug in a mice melanoma model. The mice were received three intravenous doses of EPA (60 µg/mouse) with four-day intervals started on day 10 accordinng to the MTT and MTD results in this and our previous study^29^, and three subcutaneous doses of gp100 melanoma vaccine (25 µg/mouse) with seven-day intervals started on day 7. The treatment protocol was selected according to the objectives of the study, studying the effect of combination of a drug and a vaccine. As the vaccine’s effect will be assessable after about a week, we scheduled the treatment protocol in this way, so that at the end of the study, we can evaluate the effect of both interventions, namely the drug and the vaccine. Similarly, Chen and colleagues have used Epacadostat with four-day intervals^30^.

In the first step of our study, we evaluated IDO expression in our cell lines. The results of PCR showed relative IDO expression in the B16F10 melanoma tumor. These results are consistent with the previous evidence on the (T cell dependent) immunosupresive role of IDO1 in melanoma and its effect on survival, which resulted in suggestion of IDO1 inhibitors as a promising cancer treatment target^47–49^. EPA, a selective and reversible inhibitor of IDO1, competeitvely blocks tryptophan catbolism by IDO and increases the proliferation of CD8^+^ T and natural killer (NK) cells, production of IFN-γ, and supression of Tregs^50^. Also, the reduced mRNA expression of the IDO gene in all therapeutic groups of our study, compared to the PBS control group, confirms the protective immune responses of all therapy groups.

Although in our results, there was no significant difference in IDO gene expression level in the treated mice groups, the lowest expression level of this enzyme was observed in the liposomal combination therapy group which indirectly affected the more favorable immune responses in the liposomal combination therapy group. We also observed a difference in the level of immune responses between the two groups of free and liposomal epacadostat.

Although in vitro studies have shown EPA, a highly selective IDO1 inhibitor with moderate oral bioactivity, results in increased activity of cytotoxic T lymphocyte^51,52^, in vivo studies (phase III clinical trials) have failed to show significant response to treatment after application of EPA or its superiority to pembrolizumab, which could be because of the limited capacity for IDO1 inhibition at the tumor site^53,54^. Nanotechnology has introduced lipid-based nanoparticles, particularly liposomes, with satisfactory results for delivery of the drug to the tumor area, reduction of adverse effects, and increased solubality of the hydrophobic drugs^55^. Accordingly, we have designed a PEGylated liposome for delivery of EPA specifically to the tumor site and the results of this method showed the successful loading of EPA on liposomes as describued previously^29^. This method has the advantage of not being affected by dilution factor and higher stability of the drug. These results showed that the remote loading of EPA using ammonium sulfate gradient into the core of liposomes increased the stability of the drug. These results are consistent with the previous studies of high efficacy of remote loaded liposomes^56^.

We observed the maximum accumulation of liposomal EPA at tumor site 48 h after injection and 3.12 µg liposomal gp100 vaccine (FL1) formulation was qualified as favorable formulation for the rest of the investigations. These results show that labeling of liposomal EPA resulted in succesful accumulation of the drug in the tumor area, resolved the major drawback for using this potent IDO inhibitor. Other preclinical studies have also confirmed the successful loading of drugs and vaccines on liposome nanoparticles^55,57^, while the selected drug/vaccine and liposomes differ from that in the present study. Furthermore, the previous clinical trials failed to show superiority of the combination of EPA with other immunotherapy agents, compared to monotherapy^58,59^, while we showed the superiority of the suggested liposomal combination to each of the therapies alone in the following aspects.

In addition to EPA, Lip-gp100 has been also suggested as an efficient immunosuppressant in melanoma. In a study on mice inoculated with the same cell line as in the present study, using of cationic liposomes with gp100_25-33_ self-antigen and comparsion with its free form showed a favorable tumor regression for the liposomal form^40^, which confirms the general results of the present study, considering the effectiveness of Lip-gp100 as a vaccine for melanoma. The results of the present study are also in line with the results, suggesting favorable treatment of melanoma with gp100 in combination with other immunotherapeutic agents^60^, although they were not liposome-loaded and not combined with the agents similar to the present study.

The lowest tumor growth rate in the liposomal combination therapy group (Lip-EPA + Lip-gp100) compared to the other groups confirmed its efficacy. Also, the combination therapy group had the lowest incidence of death, highest TTE, MST, ILS, and TGD with a significant difference in TTE, TGD, and ILS between non-liposomal and liposomal treatment groups. These results are in line with those obtained by clinical trials, indicating promising results for EPA on the survival rate of patients^61,62^ and confirms the favorable effect of EPA on preclinical outcomes, although the previous reports failed to show additional effect for the combination of EPA with other agents^58,59^. However, we observed the significant superiority of the liposomal combination therapy group (Lip-EPA + Lip-gp100) over other groups.

Considering the fact that IDO1 overexpression in melanoma cells upregulates Tregs and impairs the function of CD8^+^ and CD4^+^ T cells^63^, EPA has an impact role on activation of human tumor antigen-specific cytotoxic T cell line, proliferation of CD8^+^ T and NK cells, production of IFN-γ, and supression of Tregs^10^. Therefore, we performed specific evaluation of the tissues (lymphocytes in the spleen, lymph node, and tumor infiltrated cells) using flow cytometric analysis in the treated mice, in order to identify the potential of the different therapies on tumor regression. A higher level of CD8^+^ T cells, Th1 (CD3^+^CD4^+^ T cells), and IFN-γ production, as well as lower Th2 (CD3^+^CD4^+^ T cells producing IL-4) and Treg cells (CD4^+^CD25^+^FOXP3^+^ T cells producing IL-10) were observed in the liposomal combination therapy group (Lip-EPA + Lip-gp100), compared to the other groups. Evidence has outlined the role of immunodeficiency in pathogenesis of advanced melanoma, induced by a specific disruption of genes affecting critical the immune system components, producing or inhibiting several cytokines^64^. Lymphocytes include CD4^+^ and CD8^+^ T cells, and B-cells. The CD4^+^ T cells, which includes two types of Th1 (induced by production of IFN-γ) and Th2 (responsible for B-cell antibody secretion, including interlukins) plays the major role of the immune response in melanoma, including the stimulation of CD8 + T cells activity by mediators such as IFN-γ, or IL-2^65,66^. Previous studies have also shown the anti-tumour immunity, induced by vaccines, through CD4^+^ T cell response in melanoma^67–69^. Accordingly, the increased CD4^+^ and CD8^+^ T cells, Th1, and IFN-γ production in the liposomal combination therapy group in the present study showed the greater efficacy of this method in tumor suppression. On the other hand, Th2 has been identified to destabilize the anticancer effects of Th1 and the significantly lower Th2 in this group also confirms the anticancer efficacy of this treatment. The results of the present study are in line with the previous results on the immunostimulatory effect of EPA on CD4^+^ and CD8^+^ T cells^70^. In addition, we showed that this combination had even more effect, compared to each of the treatments alone. The combination of pH-sensitive liposomal dual-delivery system for EPA and doxorubicin was also identified effective on suppression of lung metastasis of melanoma through activation CD8^+^ cytotoxic T lymphocytes^30^, which confirms our results of the present study, although their formulations were different.

Another important immune regulatory cell in melanoma and other cancers is Treg cells (CD4^+^CD25^+^ regulatory T cells), which have an immunosuppressive function by increased expression of CTLA-4 and PD-1, resulting in suppression of antitumor immunity^71^. The significantly lower Treg level in the liposomal combination therapy group confirmed the previous results considering the efficacy of this treatment. These results are consistent with those of previous studies, which indicated that EPA decreased Treg proliferation, induced by IDO production in melanoma^10,70^. Furthermore, the cytotoxic activity of CTL cells in this group was higher than the other groups. Chen and colleagues also showed the activated T cell mediated cytotoxicity by pH-sensitive liposomal dual-delivery system for EPA and doxorubicin^30^. Yazdani and colleagues also confirmed the highest cytoxic activity and IFN-γ production in tumor infiltrated lymphocytes at the tumor area, indicated by the gp100 liposomal vaccine^40^. Although the results obtained in the present study are in line with the previous reports on efficacy of EPA^30^ or gp100 vaccine^40^ in cytotoxic activity of CTL cells in melanoma, a significantly higher effect of the combination therapy group in liposomal form (i.e. Lip-EPA + Lip-gp100) was the new finding of our study.

## Conclusion

Our results showed that the combination of Lip-EPA and Lip-gp100 was significant on IDO1 inhibition, the immunostimulatory effects, as well as preclinical outcomes, including survival and tumor growth rate. These results suggest this combination as an effective treatment with capability of being used in prophylactic and therapeutic studies that merits further investigations. The combination of gp100 peptide (CTL specific epitope) in the form of liposomal vaccine with liposomal epacadostate (modulator of immunosuppression in TME) exhibited the desired improvements compared to non-liposomal combination therapy but still can be improved. Addition of immunoadjuvants like MPL, CpG, etc. plays an important role in induction of immune. Furthermore, the use of a CD4^+^ T cell activator peptides like PADRE, can induce CD4^+^ T cells activity which enhance the antitumor activity of CD8^+^ T cells and anti-cancer vaccine's efficiency.

## Supplementary Information

Supplementary Information.

## Data Availability

The data that support the findings of this study will be made available upon reasonable request. The request should be send to Dr Alireza Rafiei.
