# Supplementary material for: Targeting the tumor microenvironment by liposomal Epacadostat in combination with liposomal gp100 vaccine
Source: Sci Rep. 2023 Apr 10;13:5802. doi: 10.1038/s41598-023-31007-x (PMC10086071; doi:10.1038/s41598-023-31007-x)

**Targeting the Tumor Microenvironment by Liposomal Epacadostat in combination with Liposomal gp100 Vaccine**

Sahar Tahaghoghi-Hajghorbani a, b, Mona Yazdani b, Amin Reza Nikpoor c, Mahdi Hatamipour ^b^, Abolghasem Ajami ^a^, Mahmoud Reza Jaafari b, d, e, Ali Badiee b, e **, Alireza Rafiei f *

a Department of Immunology, School of Medicine, Mazandaran University of Medical Sciences, Sari, Iran

^b^ Nanotechnology Research Center, Pharmaceutical Technology Institute, Mashhad University of Medical Sciences, Mashhad, Iran

^c^ Department of Immunology, Faculty of Medicine, Hormozgan University of Medical Sciences, Bandar Abbas, Iran

^d^ Biotechnology Research Center, Pharmaceutical Technology Institute, Mashhad University of Medical Sciences, Mashhad, Iran

^e^ Department of Pharmaceutical Nanotechnology, School of Pharmacy, Mashhad University of Medical Sciences, Mashhad, Iran

^f^ Department of Immunology, Molecular and Cell Biology Research Center, School of Medicine, Mazandaran University of Medical Sciences, Sari, Iran

* **Corresponding author:**

Alireza Rafiei (Ph. D.)

Professor in Immunology,

Department of Immunology, Molecular and Cell Biology Research Center, School of Medicine, Mazandaran University of Medical Sciences, Sari, Iran

Phone: +98-11-33543088

Fax: +98-11-3354-3087

** **Co-corresponding author:**

Ali Badiee (Pharm. D., Ph. D.)

Associate Professor in Pharmaceutics,

School of Pharmacy, Mashhad University of Medical Sciences, Mashhad, Iran

P.O. Box: 91775-1365

Phone: 0098-513-1801339 (Office)


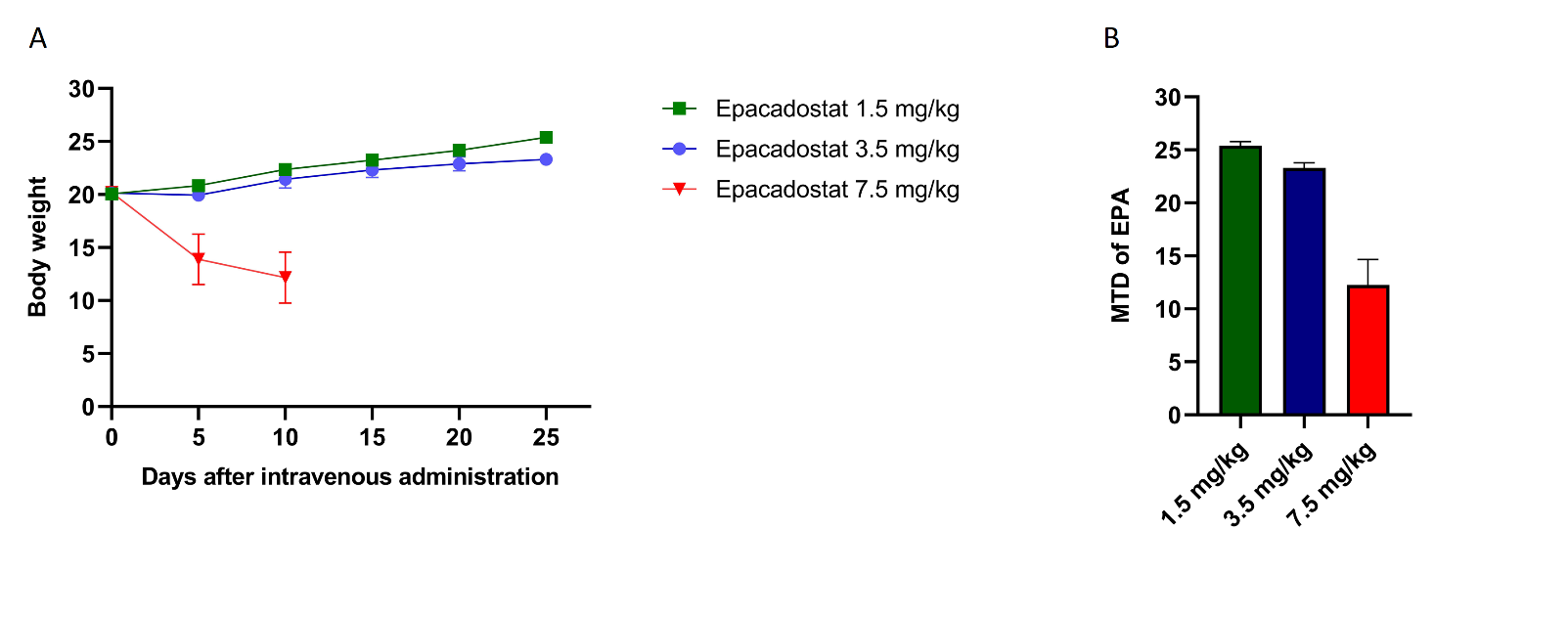


**Figure S1. Maximum tolerated dose (MTD)** **of Epacadostat (EPA) in C57BL/6 mice.** (A) Mice body weight changes after two administrations of three different doses of Epacadostat. (B) The MTD of EPA. Data are expressed as mean ± SD (n = 6).

**Gating Strategy in Lymph Node and Spleen**


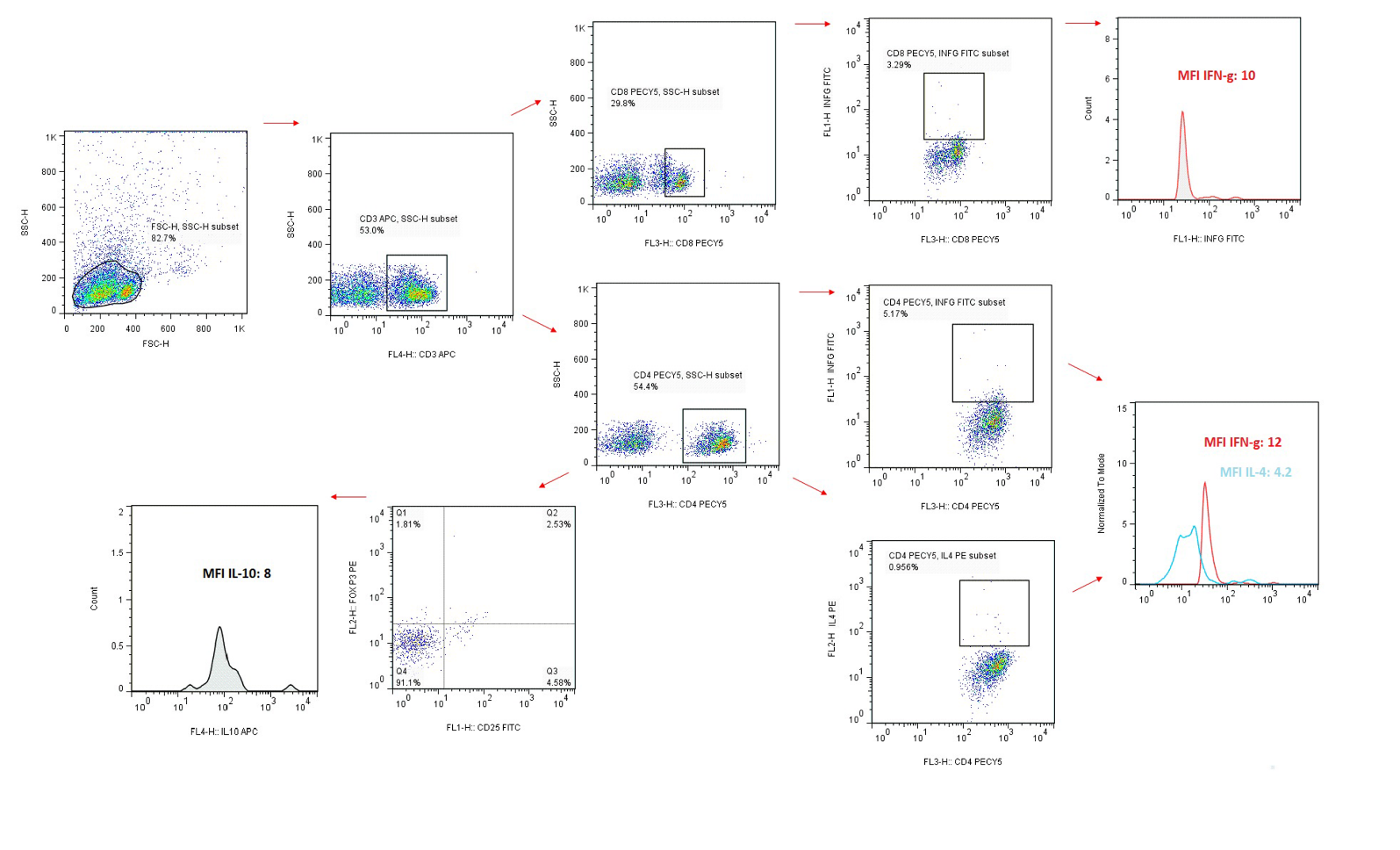


**Gating Strategy in Tumor**


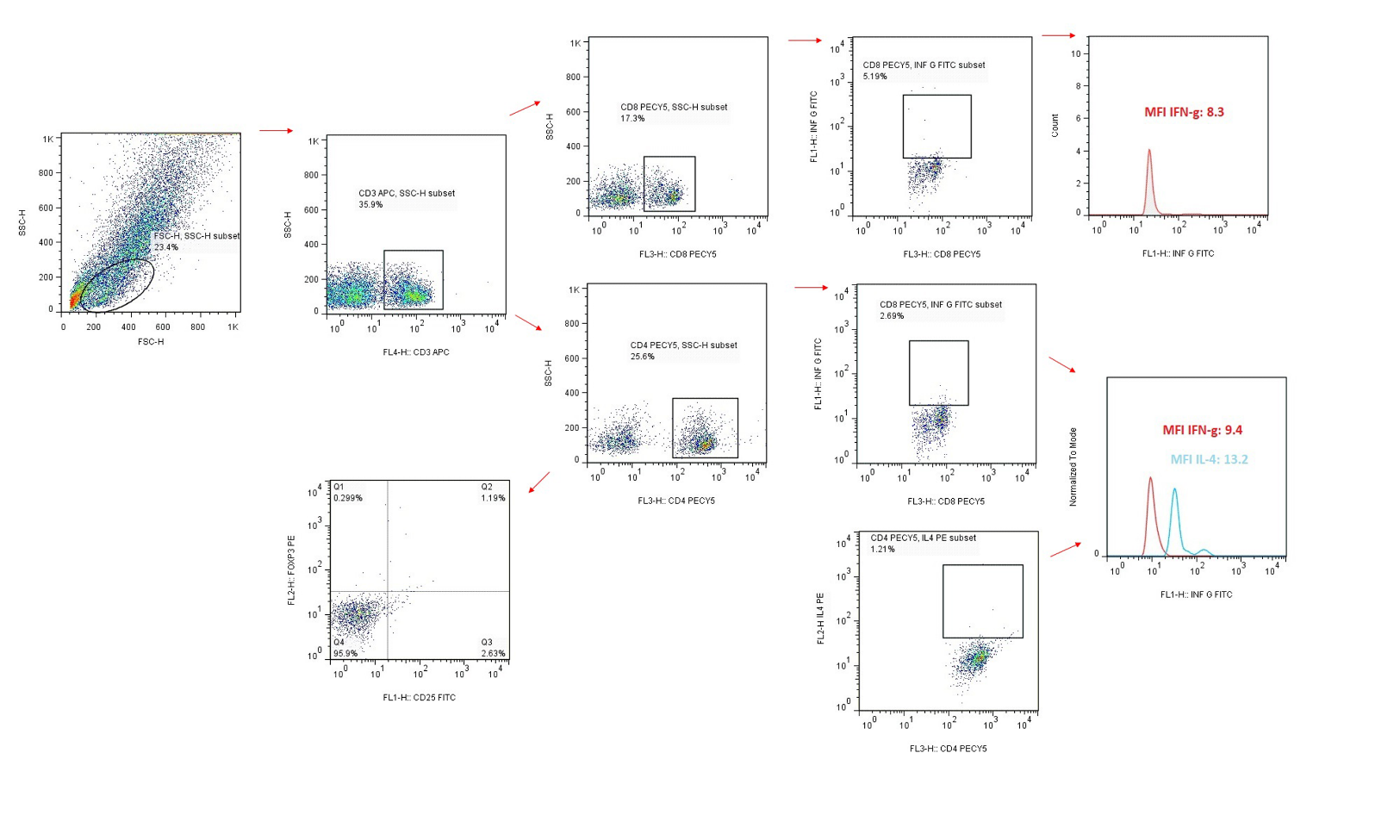


**Figure S2: Representative dot plots of CD8, CD4 and Tregs in** **different tissues.** Lymph node, Spleen and tumor tissues obtained from different groups were stained with fluorochrome-labeled antibodies against CD3, CD4, CD8, CD25 and FoxP3. For CD8+ and CD4+ T cells, acquired cells were first gated on CD3+ T cells followed by gating for CD4+ or CD8+ cells within CD3+ T cells for next analysis, percentage of cytokine producing cells and mean fluorescence intensity. The cells expressed CD25+ FoxP3+ within CD4+ T cells was determined as Tregs.

**Table S1**

**Statistical analysis of tumor growth and survival in mice received with different treatments.**

| **Day** | **Treatment groups** | **P-value** |
| --- | --- | --- |
| **Tumor Growth Curve** | | |
| 19, 22, 25 | Lip-EPA+Lip-gp100 *vs*  all groups except Lip-gp100 | p < 0.0001 |
| 28 | Lip-EPA+Lip-gp100 *vs*  all groups | p < 0.0001 |
| **Survival** | | |
| 28 | Lip-EPA+Lip-gp100 *vs*  EPA and Lip-EPA | p < 0.01 |
|  | Lip-EPA+Lip-gp100 *vs*  PBS | p < 0.001 |

**Table S2**

**Statistical analysis of TTE, TGD and ILS in different treatment groups.**

|  | **Treatment groups** | **P-value** |
| --- | --- | --- |
| **TTE** | EPA *vs* EPA+gp100 and Lip-EPA+Lip-gp100 | p < 0.0001 |
|  | gp100 *vs* Lip-gp100, EPA+gp100  Lip-EPA+Lip-gp100 | p < 0.0001 |
|  | Lip-EPA *vs* EPA+gp100 | p < 0.001 |
|  | Lip-EPA *vs* Lip-EPA+Lip-gp100 | p < 0.0001 |
|  | Lip-gp100 *vs* EPA+gp100 | ns |
|  | Lip-gp100 *vs* Lip-EPA+Lip-gp100 | p < 0.01 |
|  | EPA+gp100 *vs* Lip-EPA+Lip-gp100 | p < 0.01 |
| **TGD** | EPA *vs* Lip-EPA | p < 0.01 |
|  | EPA *vs* EPA+gp100 and Lip-EPA+Lip-gp100 | p < 0.0001 |
|  | gp100 *vs* Lip-gp100, EPA+gp100  Lip-EPA+Lip-gp100 | p < 0.0001 |
|  | Lip-EPA *vs* EPA+gp100 and Lip-EPA+Lip-gp100 | p < 0.0001 |
|  | Lip-gp100 *vs* EPA+gp100 | ns |
|  | Lip-gp100 *vs* Lip-EPA+Lip-gp100 | p < 0.0001 |
|  | EPA+gp100 *vs* Lip-EPA+Lip-gp100 | p < 0.0001 |
| **ILS** | EPA *vs* Lip-EPA | ns |
|  | EPA *vs* EPA+gp100 and Lip-EPA+Lip-gp100 | p < 0.0001 |
|  | gp100 *vs* Lip-gp100, EPA+gp100  Lip-EPA+Lip-gp100 | p < 0.0001 |
|  | Lip-EPA *vs* EPA+gp100 and Lip-EPA+Lip-gp100 | p < 0.0001 |
|  | Lip-gp100 *vs* EPA+gp100 and Lip-EPA+Lip-gp100 | p < 0.0001 |
|  | EPA+gp100 *vs* Lip-EPA+Lip-gp100 | p < 0.01 |

**Figure 2 original image**


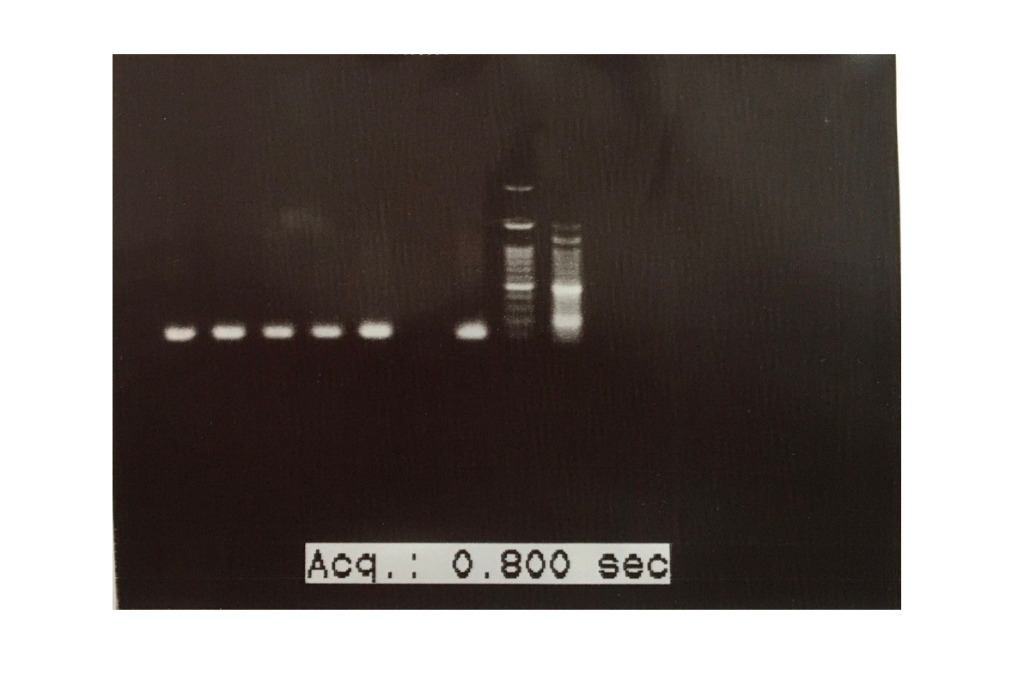


**Figure 7**

GAPDH Gel


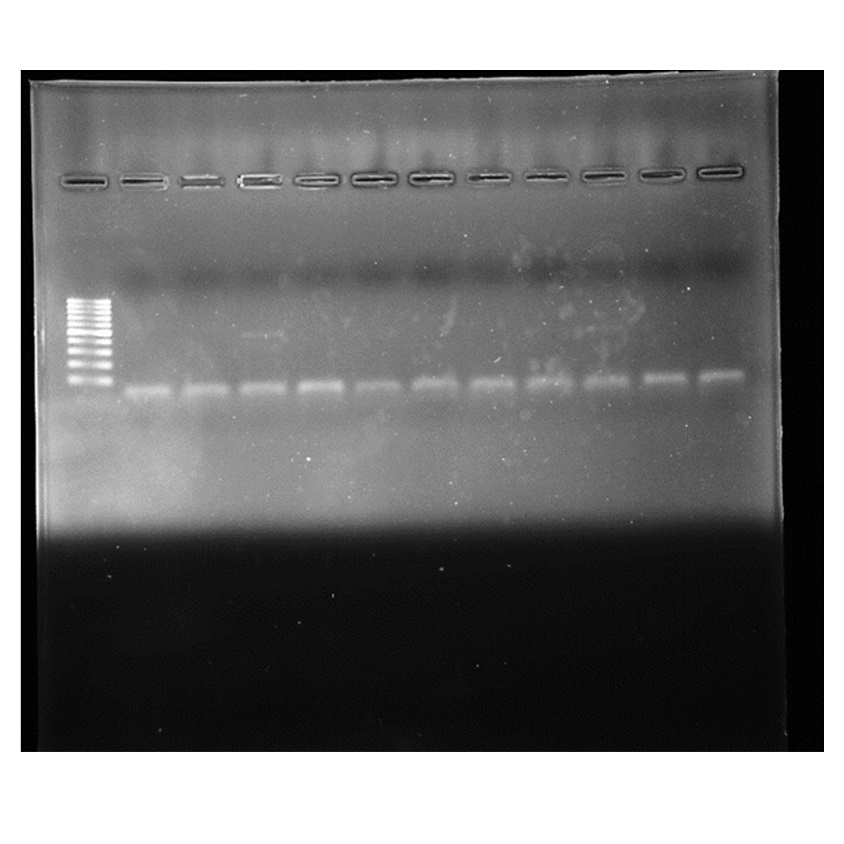


IDO Gel


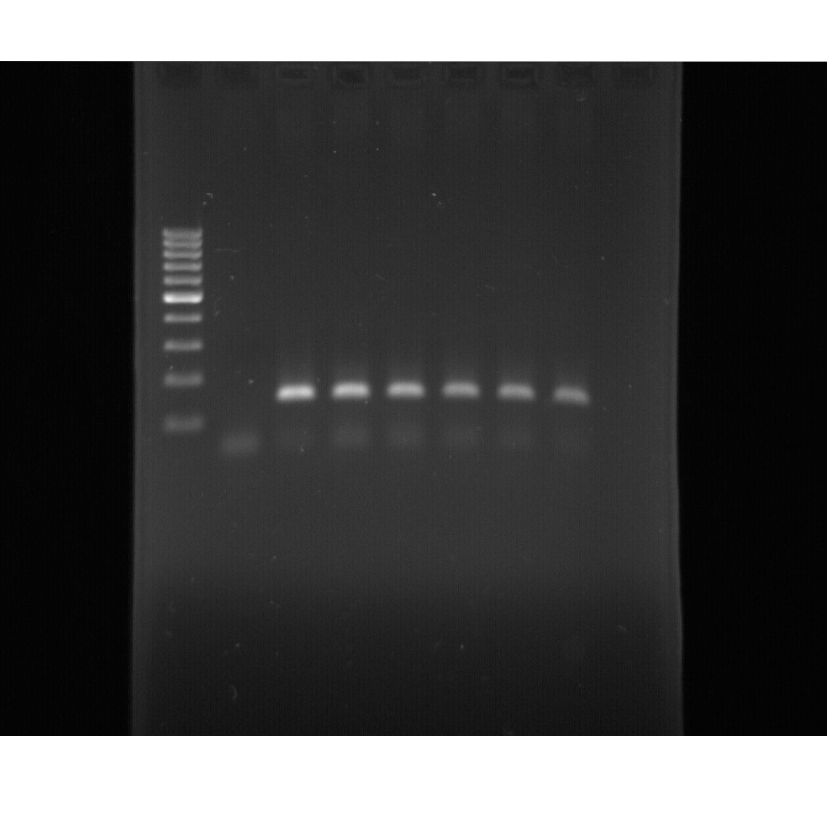

Supplement: Supplementary file 1 — Supplementary Information. [file 41598_2023_31007_MOESM1_ESM.docx]
